# Exploring the Link: Unraveling the Connection Between Hearing Loss and Psychiatric Disorders

**DOI:** 10.7759/cureus.83223

**Published:** 2025-04-29

**Authors:** Dana J Al Tarawneh, Yusuf J Al Tarawneh, Abdallah Khan, Mohammed Abdul Muqsit Khan, Tabish W Siddiqui, Raqshan W Siddiqui, Syed Muhammad Hayyan Nishat, Asma A Alzaabi, Fatema M Alzaabi, Shiza W Siddiqui

**Affiliations:** 1 Internal Medicine, RAK Medical and Health Sciences University, Ras Al Khaimah, ARE; 2 Internal Medicine, King Khalid University Hospital, Abha, SAU

**Keywords:** cognitive impairment and dementia, depression, hearing aid, illness anxiety disorder, psychiatric disorders, psychosis, hearing loss

## Abstract

Hearing loss is a prevalent condition, particularly in older adults, with significant implications for mental health. This review explores the complex relationship between hearing impairment and psychiatric illnesses, including cognitive decline, depression, anxiety, and psychotic symptoms, through an analysis of current literature. This work highlights the dual impact of untreated hearing loss on auditory function and mental well-being by examining cross-sectional studies, case-control studies, retrospective studies, and meta-analyses. Key findings include the association of hearing loss with increased risks of dementia, social isolation, and mood disorders, driven by heightened cognitive load and communication challenges. Interventions such as hearing aids, cochlear implants, psychotherapy, and pharmacological treatments demonstrate the potential to alleviate these risks and improve mental health outcomes. However, their utilization remains limited, particularly in underserved regions. This underscores the need for early detection, multidisciplinary management, and expanded access to auditory care. By synthesizing evidence across disciplines, this review offers new insights into the interplay between hearing impairment and psychiatric health, informing strategies for integrated intervention. Addressing hearing loss is crucial for improving communication and mitigating its profound effects on mental health, thereby enhancing quality of life across populations.

## Introduction and background

Hearing loss is a common and often progressive condition, particularly in older adults, with significant implications for communication, quality of life, and financial stability [1]. Globally, it affects 6.8% of the population, with a higher prevalence in men and increasing incidence with age [2]. Daily interactions become difficult for those with moderate-to-severe hearing loss, which frequently results in social isolation [2]. The possibility that this isolation could worsen or cause cognitive deterioration makes it worrisome [1]. Therefore, the consequences of hearing loss affect many areas of life, not just auditory issues [1].

Despite its high prevalence and broad impact, hearing loss is often overlooked in mental health settings [1]. The growing burden of age-related hearing loss (ARHL) in the global population makes it critical to explore its full spectrum of clinical consequences, including psychiatric comorbidities and cognitive decline [1].

ARHL is a prevalent impairment that often remains underdiagnosed and underestimated in terms of its impact on physical and mental well-being [1]. It is defined by measuring the strength of sound required for an individual to perceive noises at different frequencies, which are essential for interpreting speech and going beyond subjective complaints [1]. This evaluation usually covers frequencies between 250 and 8,000 Hz, with decibels (dB), a logarithmic scale that reflects sound intensity, used to test hearing thresholds [1]. Adults with normal hearing can distinguish between sounds as low as 0 and 20 dB, which includes sounds as subtle as a person's breath [1]. Different thresholds are displayed by people who have hearing loss: mild (21-40 dB), moderate (41-55 dB), fairly severe (56-70 dB), severe (71-90 dB), and profound (beyond 90 dB). These thresholds impact an individual's capacity to perceive sound at different levels, ranging from whispers to loud sounds [1,2].

History and examination should be performed [1]. The clinician needs to note if one or both ears are affected, the rate of onset, previous employment, history of ingestion of any potentially ototoxic drugs, and any of the major symptoms of ear disease [1]. These are pain (otalgia), discharge (otorrhea), a sensation of abnormal movement (vertigo), and inappropriate noise in the ear (tinnitus) [1]. It is important to rule out dementing and affective disorders since confusion and inattention may be misinterpreted as evidence of hearing impairment [1]. Audiologists administer hearing tests using electronic equipment [1]. In pure-tone audiometry, tones of different frequencies are presented at various intensities to each ear via bone and air conduction [1]. The patient signals when they become aware of the tone [1]. An audiogram can be plotted to show the threshold for each frequency [1]. Pure-tone audiometry can determine the severity of hearing loss and identify conductive loss or a conductive component [1].

Recent studies suggest that factors associated with mental health conditions, such as hearing loss, may be modifiable [3]. According to this, preventative measures and treatments can lessen the negative impacts of hearing loss and enhance general public health [3]. Effectively managing hearing loss may help lower related risks, such as social isolation and cognitive decline, providing a means of improving one's physical and mental health [3]. Interventions might be anything from encouraging early identification and management techniques to expanding access to hearing aids [3]. Furthermore, research suggests that compared to the general population, those with hearing impairments have greater anxiety rates [4]. The degree of hearing impairment is correlated with this elevated anxiety, which may subside after surgical intervention [4]. In general, hearing loss has significant ramifications while being a common and frequently progressive disorder [4]. Improving communication, reducing its consequences, and raising the quality of life all depend on efficient management and prompt intervention [4]. In addition to assisting with the immediate effects of hearing loss, treating it may also help avoid related hazards, promoting a more cohesive and healthy community [4].

More and more research has shown that hearing loss is a major risk factor for several mental health conditions, especially in older adults [5]. Understanding the relationship between mental health and hearing loss becomes crucial as the world's population ages to enhance patient care and general quality of life [1]. Research constantly demonstrates an association between psychological disorders such as psychosis, depression, anxiety, and cognitive decline, including dementia, and hearing impairment [1].

Although these associations are well-documented, the need remains for a consolidated review that synthesizes current findings, highlights mechanisms underlying these relationships, and evaluates the effectiveness of treatment strategies. The relationship between hearing loss and mental health risks is reviewed in this work, along with its underlying mechanisms, the psychosocial effects of hearing loss, and the effectiveness of therapies targeted at reducing these risks [1].

In this article, we present a systematic review of the relationship between hearing loss and psychiatric illness by analyzing cross-sectional studies, case-control studies, retrospective studies, and review articles. This review builds upon existing literature by offering a multidisciplinary perspective and emphasizing the importance of early diagnosis, integrated care, and access to auditory and psychiatric interventions, particularly in aging and underserved populations.

## Review

Methodology

Search Strategy

We conducted a systematic review following the Preferred Reporting Items for Systematic Reviews and Meta-Analyses (PRISMA) guidelines. A comprehensive literature search was performed in three major databases: PubMed, Scopus, and Google Scholar, using keywords such as “hearing loss and psychiatric disorders,” “hearing impairment and mental health,” and “auditory dysfunction and psychiatric illness.” The Boolean operator (AND) was employed to refine search results.

In addition, we used combinations of MeSH terms and free-text terms, such as (“hearing disorders” OR “hearing impairment” OR “hearing loss”) AND (“mental health” OR “psychiatric illness” OR “cognitive decline” OR “depression” OR “anxiety”), tailored to each database. Search filters were applied to include only human studies published in English between 2000 and 2024.

**Figure 1:**
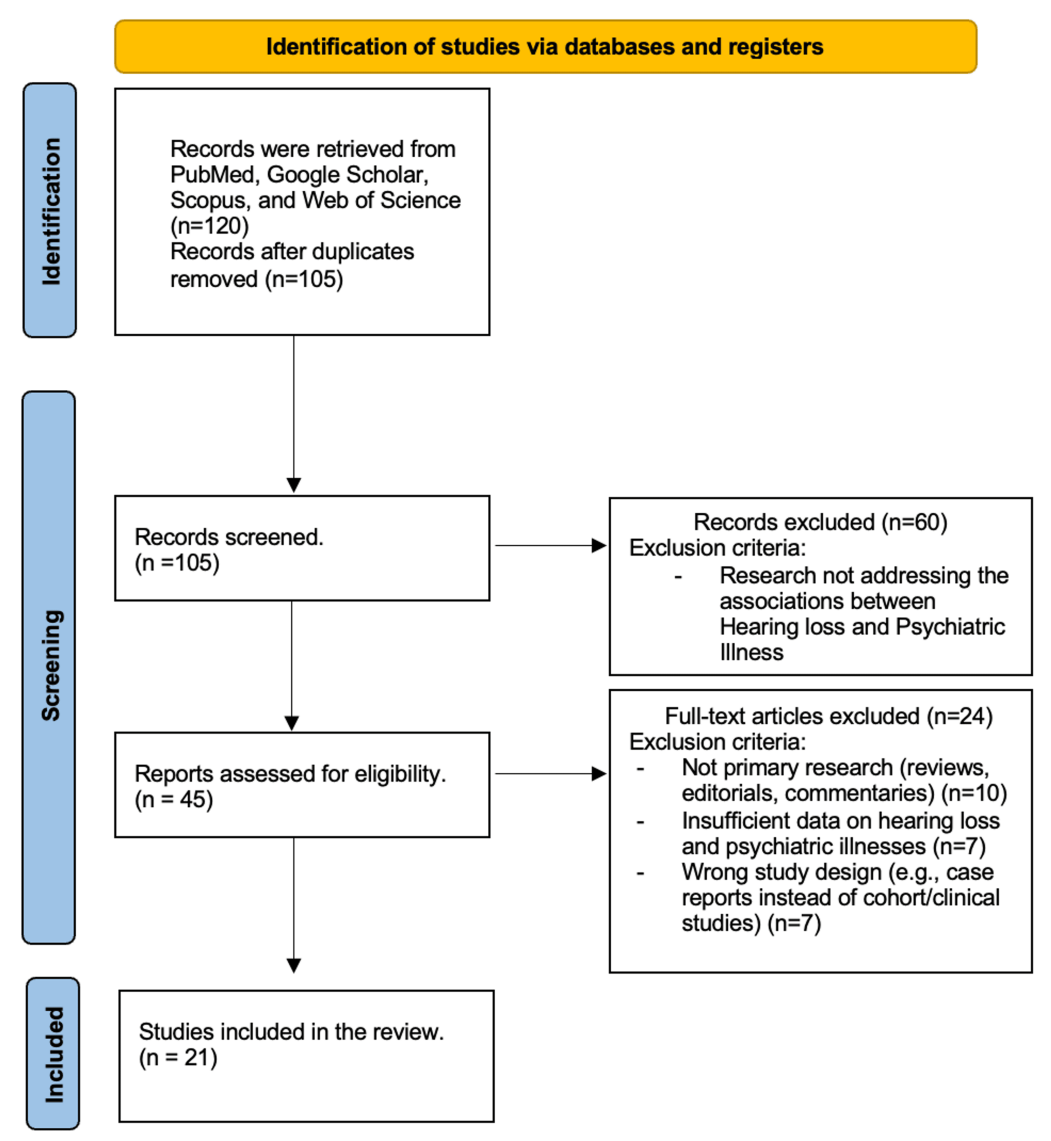
PRISMA flowchart of selected articles PRISMA: Preferred Reporting Items for Systematic Reviews and Meta-Analyses

The initial database search yielded 95 articles, and after removing duplicates, 90 records remained for screening. Titles and abstracts were screened for relevance, leading to the selection of 40 full-text articles for further assessment of eligibility. After applying the inclusion and exclusion criteria, 25 studies were included in the final synthesis. The study selection process is visually outlined in the PRISMA flowchart (Figure 1).

Two independent reviewers screened titles, abstracts, and full texts for eligibility. Discrepancies were resolved through discussion or consultation with a third reviewer. Data were extracted using a standardized form that captured the study design, sample size, population characteristics, measures of hearing loss, psychiatric outcomes assessed, and key findings.

Given the diversity in study designs, outcome measures, assessment tools, and populations across the included studies, a meta-analysis was not conducted. These methodological and clinical differences would have made statistical pooling inappropriate and potentially misleading. Therefore, a narrative synthesis was used to integrate the findings. This approach allows for a comprehensive, flexible review of the evidence while addressing the complexity and multidimensional nature of the association between hearing loss and psychiatric disorders.

Inclusion and Exclusion Criteria

The inclusion criteria targeted observational studies (cross-sectional, cohort, and meta-analyses) that examined the association between hearing loss and psychiatric disorders. Studies providing statistical analyses of this relationship, along with those reporting on the prevalence, risk factors, and potential mechanisms linking hearing impairment to psychiatric illnesses, were included. Only articles published in peer-reviewed journals were considered. The exclusion criteria followed PRISMA recommendations and removed studies that did not focus on hearing loss and psychiatric illness, including case reports, editorials, and reviews without primary data. Qualitative studies lacking statistical analyses were also excluded. Additionally, full-text articles were excluded if they lacked statistical analysis of the association (n = 15), had small sample sizes or lacked representativeness, or contained incomplete or unclear data. To assess study quality and risk of bias, we used the Newcastle-Ottawa Scale (NOS) for observational studies and the AMSTAR-2 tool for meta-analyses. Studies were categorized as high-, moderate-, or low-quality based on these assessments, and only moderate-to-high-quality studies were included in the final review.

Results

The initial search identified 95 articles across three databases. After removing duplicates, 90 articles underwent title and abstract screening, and 50 articles were excluded for irrelevance or failure to meet the inclusion criteria. The remaining 40 full-text articles were assessed, with 15 being excluded due to the lack of statistical analysis, small sample size, or incomplete data. Ultimately, 25 studies were included in the final synthesis. The detailed selection process is illustrated in the PRISMA flowchart (Figure 1).

The included studies varied in design, with most being cross-sectional and cohort studies and a few meta-analyses. Across these studies, there was a consistent association reported between hearing loss and increased prevalence of psychiatric conditions, particularly depression, anxiety, and cognitive decline. Several studies also noted that the severity of hearing loss positively correlated with mental health burden.

Despite some variation in populations studied (e.g., age groups, care settings, and geographic regions), a recurring pattern was that untreated or unrecognized hearing impairment often led to social isolation, reduced cognitive stimulation, and emotional distress, all of which contributed to poor mental health outcomes.

However, the heterogeneity in study design, diagnostic criteria for both hearing loss and psychiatric disorders, and methods of outcome measurement introduces some limitations in comparing findings across studies. Some studies used self-reported questionnaires while others used clinical diagnosis or audiometry, which may affect consistency. Nonetheless, the overall trend remained robust: interventions like hearing aids and cochlear implants were frequently associated with improved mental health outcomes, though underutilization was a common theme across most settings.

These findings underscore the need for standardized approaches in future research to enable clearer comparisons and meta-analytic synthesis. The results support the hypothesis that hearing loss is not only an auditory issue but also a substantial contributor to psychiatric morbidity.

Discussion

Hearing loss is increasingly recognized as a significant factor contributing to various psychiatric disorders, with complex mechanisms linking the two [2]. Hearing impairment can exacerbate mental health issues through social isolation, communication difficulties, and cognitive strain [2]. These challenges often lead to feelings of loneliness, depression, and anxiety, as individuals struggle to engage in conversations and process auditory information [2]. Additionally, hearing loss can accelerate cognitive decline, increasing the risk of dementia, and may contribute to psychotic symptoms, such as auditory hallucinations, in severe cases [2]. This underscores the importance of early detection and intervention for hearing loss to prevent its psychiatric consequences [2]. In the following discussion, we will explore the relationship between hearing loss and specific psychiatric disorders, including depression, anxiety, cognitive decline, and psychosis.

Hearing Loss and Cognitive Decline

The correlation between hearing loss and mental health is particularly noteworthy because of its role in dementia and cognitive decline [5]. Presbycusis, ARHL, is a common condition among the elderly and has recently been recognized as a risk factor for dementia and is estimated to account for up to 9.1% of the modifiable risk for this disease [5]. However, hearing loss is one of the most unrecognized deficits in subjects with Alzheimer's disease [5]. According to studies, hearing loss not only lowers auditory input but also puts additional cognitive stress on the brain by requiring it to focus more of its resources on interpreting distorted sound, which impairs memory and executive function, among other cognitive functions [4,5].

According to research, the connection between ARHL and dementia involves multiple, interrelated mechanisms [6]. One primary pathway is the cognitive load hypothesis, which suggests that ARHL increases the effort required for auditory processing [6]. As auditory input decreases, the brain reallocates resources to decode and interpret sounds, leaving fewer cognitive resources available for other essential functions, such as memory and executive tasks [6]. Over time, this chronic cognitive strain can accelerate cognitive decline, predisposing individuals to dementia [6].

Another significant mechanism is auditory deprivation, which leads to structural and functional changes in the brain [7]. Reduced stimulation of the auditory pathways can result in atrophy of brain regions involved in auditory processing, such as the temporal lobe, and those critical for broader cognitive functions [6]. Neuroimaging studies have demonstrated that individuals with ARHL experience faster rates of brain volume reduction, particularly in areas crucial for language and memory [6]. This physical decline in brain structure may underlie the increased risk of dementia observed in individuals with ARHL [7].

Social isolation and reduced cognitive stimulation also play a critical role in the ARHL-dementia link. Hearing loss can create communication barriers, leading to social withdrawal and loneliness [6]. The lack of social interaction deprives individuals of cognitive engagement, a protective factor against cognitive decline [6]. Moreover, social isolation is associated with an increased risk of depression, which is itself a known risk factor for dementia [6]. These compounding effects highlight the indirect but powerful influence of ARHL on cognitive health [6].

ARHL and dementia may also share common biological pathways, such as vascular changes, chronic inflammation, and oxidative stress [6]. These systemic processes can simultaneously damage the auditory and cognitive systems, suggesting a shared pathophysiology that increases the risk of both conditions [6]. Furthermore, ARHL often coexists with age-related comorbidities like hypertension, diabetes, and cardiovascular disease, which are independently linked to dementia [6]. The combined effects of ARHL and these conditions may amplify the overall risk of cognitive decline [8].

Together, these mechanisms underscore the complex interplay between ARHL and dementia, involving direct neural impacts, increased cognitive burden, and indirect effects through social and systemic factors [7]. This understanding emphasizes the importance of early intervention and management of ARHL as a potential strategy to mitigate the risk of dementia [9].

With appropriate management, it may be possible to prevent approximately 900,000 cases of dementia each year [10]. These results highlight the necessity of treating hearing impairment as soon as possible to prevent cognitive decline from getting worse [10].

It has been demonstrated that interventions like cochlear implants and hearing aids may reduce the adverse impact of hearing loss on cognitive decline [5]. It was shown by research that studied the effect of cochlear implantation on cognitive decline and quality of life in older adults that after implantation, adult patients demonstrated improved cognition, speech perception, and quality of life [7]. By strengthening auditory function, these gadgets reduce cognitive strain and increase mental clarity [5]. As a result, the rate of cognitive decline is slowed, improving the quality of life for those who have hearing loss. But despite their proven benefits, hardly enough people wear hearing aids, especially for the most vulnerable, including residents of nursing homes [11]. There are several reasons for this, the first of which is that many residents' hearing loss has gone unnoticed [11]. For instance, it is possible to infer that dementia, rather than a hearing impairment, is the cause of a person's incapacity to comprehend what is being said [11]. Both determining the extent of hearing loss and fitting hearing aids for persons with dementia may be challenging [11]. Once provided, hearing aids may provide challenges for use, such as poor fit, improper volume control, dead batteries, and the potential for loss or breakage [11]. The majority of residents require assistance wearing their hearing aids, but staff members sometimes lack the confidence to do so or just do not believe the effort is worth making [11].

Recent findings further underscore the significance of addressing hearing impairment in aging populations [12]. A longitudinal study demonstrated that self-reported hearing loss is a strong predictor of a five-year decline in higher-level functional capacity, particularly in domains related to social participation and communication [12]. These results suggest that untreated hearing loss may exacerbate cognitive and social deterioration, emphasizing the critical need for early detection and intervention [12]. Given the established benefits of hearing aids and cochlear implants in mitigating cognitive decline, increased efforts are necessary to promote their adoption, especially among vulnerable populations such as nursing home residents [12]. Addressing barriers to hearing aid use including proper assessment, fitting, and staff training may help optimize cognitive and functional outcomes for older adults with hearing impairment [12].

Research has shown a clear connection between hearing loss and cognitive decline. A study conducted by Deal et al. found that hearing impairment significantly increases the risk of cognitive deterioration [13]. Notably, this conclusion was reached through the use of different, independent methods, underscoring the reliability and consistency of the results [13]. Deal et al. measured hearing loss using audiometric tests as part of the Baltimore Longitudinal Study of Aging [13]. Table 1 summarizes key studies providing literature evidence on the association between hearing loss and cognitive decline in different studies. However, while the methodology used in Deal et al.'s study strengthens the findings, the relatively small sample size and pilot nature of the study may limit the generalizability of its results. Larger, longitudinal studies are needed to confirm these associations and clarify causal mechanisms.

**Table 1:** Association between hearing loss and cognitive decline in different studies. DSST: Digit Symbol Substitution Test; DWRT: Delayed Word Recall Test; 3MS: Modified Mini-Mental State Examination; PTA: pure-tone average; BIMCT: Blessed Information-Memory-Concentration Test

References	Study design	Number of participants	Dementia criteria	Hearing loss criteria	Hearing aid use	Other dementia risk factors	Conclusion
Lin et al., 2004 [14]	Prospective cohort	6,112	3MS	Pure-tone audiometry (>40 dB at 2 kHz considered hearing loss; no average taken)	Did not improve cognition	NA	Hearing impairment led to greater odds of cognitive impairment
Lin et al., 2011 [15]	Prospective cohort	639	Complete neurological and neuropsychological exam (if older than 65); BIMCT (if younger than 65)	PTA (500, 1,000, 2,000, 4,000 Hz; mild 25-40, moderate 40-70, severe >70)	No cognitive impairment	NA	Hearing loss is independently associated with incident all-cause dementia
Gurgel et al., 2014 [16]	Prospective cohort	4,463	3MS screen, followed by neuropsychologist, and geropsychiatric evaluation	Clinician ascertainment during interview	NA	APOE-E allele	Elderly individuals with hearing loss have an increased rate of developing dementia
Deal et al., 2015 [13]	Prospective cohort	253	DSST, 2013 DWRT, Incidental Learning Test, Logical Memory Test I and II, Word Fluency Test, Animals Naming Test, Boston Naming Test and Trail Making A and B, Digit Span Backward Test	PTA (500, 1,000, 2,000, 4,000 Hz; mild 25-40, moderate 40-70, severe >70)	Decreased likelihood of cognitive decline	Depression	Moderate association between moderate/severe hearing loss and memory performance

Lin et al. took a long-term approach, following participants for over a decade [14,15]. Their research not only confirmed that hearing loss is linked to cognitive decline but also suggested that not treating hearing impairment could speed up the development of dementia [14,15]. Furthermore, Gurgel et al. concluded that older adults with hearing loss exhibit a higher incidence of dementia [16]. Although these studies used robust longitudinal designs, they predominantly focused on older adult populations, which may not capture the full spectrum of age-related cognitive impacts. Additionally, the reliance on observational data in these studies limits the ability to draw definitive causal conclusions.

Their large-scale meta-analysis strengthens the case for addressing hearing loss early to protect cognitive health later in life [13-16]. Nonetheless, the heterogeneity in study populations, measures of cognitive function, and hearing loss assessment tools across the literature poses challenges in synthesizing results and drawing universal conclusions. Future research should prioritize standardized methodologies to enable clearer comparisons and more definitive insights.

Hearing Loss and Anxiety Disorders and Depression

Increased anxiety is also linked to hearing impairment [4]. People who have hearing loss frequently struggle to communicate, which makes them more socially isolated and anxious in social situations [4]. According to a systematic review, the prevalence of anxiety disorders is higher in people with hearing loss than in the general population [4]. Their psychological stress is exacerbated by this concern, which is often linked to the fear of embarrassment or misunderstanding due to their impaired hearing [4].

There seems to be a direct correlation between the degree of anxiety felt and the severity of hearing impairment [4]. Anxiety disorders are more common in those with moderate-to-severe hearing loss, especially when existing symptoms like tinnitus are present [4]. Enhanced communication, facilitated by surgical interventions or the use of hearing aids, has been shown to substantially reduce anxiety symptoms [4]. However, despite these benefits, there remains a critical need for more comprehensive mental health support for individuals with hearing loss, as hearing aids alone often do not fully alleviate symptoms of anxiety [2].

Another mental health condition that is frequently linked to hearing loss is depression [4]. A common consequence of hearing loss is social isolation, which plays a significant role in the development of depressive symptoms [4,5]. Untreated hearing loss causes people to hide from social situations because they are frustrated by their inability to communicate clearly, which exacerbates feelings of loneliness and eventually results in depression [1]. Studies show that the degree of depressive symptoms is correlated with the severity of hearing loss, with those who have more severe hearing loss reporting higher levels of depression [1,5].

A longitudinal study found that individuals with visual and hearing impairments also had significantly higher rates of depression, particularly among those with untreated hearing loss [1,2]. This shows that a decline in emotional well-being can be caused by the cumulative effect of sensory impairments, which can have a significant effect on mental health. Additionally, research indicates that as hearing loss worsens, depressive symptoms become more severe over time, highlighting the importance of early intervention [1].

It has been demonstrated that treating hearing loss with devices like hearing aids reduces depressed symptoms [5]. For example, individuals who use hearing aids report an improved quality of life and reduced symptoms of depression [5]. Access to these therapies is still restricted, though, especially in low- and middle-income nations, where untreated hearing loss has an even greater psychological cost [10].

A comparative analysis of studies on hearing loss and its psychological impact reveals notable differences and similarities in populations, methodologies, and findings [17,18]. Cetin et al. examined adults aged 21-30 with unilateral hearing loss confirmed via pure-tone audiometry, comparing 90 individuals with hearing loss to a control group of 90 healthy individuals without hearing loss [17]. Using the Beck Anxiety Inventory (BAI), they reported significantly higher anxiety levels in the hearing loss group (p < 0.05) [17]. Stubbs et al., by contrast, investigated older adults aged 50+ who self-reported hearing loss, encompassing a much larger cohort of 1,911 individuals with hearing loss compared to 32,218 without [18]. Stress levels were measured using the Perceived Stress Scale, and results indicated that hearing loss was associated with significantly higher stress levels (p < 0.05) [18]. Table 2 presents studies that evaluate hearing loss as a risk factor for anxiety and depression.

**Table 2:** Studies that evaluate hearing loss as a risk factor for anxiety and depression BAI: Beck Anxiety Inventory

Author	Age range in years	Description of hearing loss in sample	Measure hearing loss	Case	Controls	Measure mental health	Summary of results
Cetin et al., 2010 [17]	21–30 (adults)	20% mild, 27.8% moderate, 34.5% severe, 17.7% profound	Pure-tone audiometry	Adult patients with acquired unilateral hearing loss recruited from the ear, nose, and throat (ENT) department of a military hospital in Turkey (n = 90)	Healthy individuals who were admitted to the same ENT department with the case group (n = 90)	BAI (anxiety)	People with hearing loss had higher levels of anxiety than controls (p < 0.05)
Stubbs et al., 2018 [18]	>50 (adults)	Unclear	Self-reported	Adults aged 50+ who reported hearing loss (n = 1,911)	Adults aged 50+ who did not report hearing loss (n = 32,218)	Perceived Stress Scale	Hearing loss associated with higher stress levels (p < 0.05)

While Cetin et al. focused on clinically diagnosed hearing loss in young adults and its association with anxiety, Stubbs et al. extended the scope to self-reported hearing loss in an older population, emphasizing the broader impact on stress [17,18]. These studies highlight the psychological burden of hearing loss across different age groups, suggesting that mental health effects are not confined to a specific demographic [17,18]. However, the use of distinct methodologies (pure-tone audiometry versus self-reports) and psychological measures, such as anxiety inventories versus stress scales, underscores the challenge of directly comparing findings across studies [17,18]. Moreover, self-reported hearing loss may introduce bias due to underreporting or misclassification, particularly in older adults. These differences highlight the need for standardized diagnostic criteria and uniform mental health assessment tools to facilitate cross-study comparisons and develop targeted intervention strategies for individuals with hearing loss [17,18]. Future research should also explore underrepresented populations and include diverse cultural and socioeconomic backgrounds to better understand the global burden of hearing-related psychological outcomes.

Hearing Loss and Psychotic Symptoms

A growing body of evidence, including meta-analytic studies, confirms a significant association between hearing loss and psychosis. In the general adult population, hearing impairment is linked to an elevated risk of hallucinations and delusions [1]. This relationship is particularly pronounced among older adults, where hearing loss is associated with an increased likelihood of delirium and persecutory delusions [5]. For instance, individuals with hearing impairments may misinterpret environmental sounds or believe others are speaking about them, leading to heightened feelings of paranoia and distress [1].

Several mechanisms have been proposed to explain this connection [5]. Reduced auditory input disrupts neurocognitive processes as the brain attempts to compensate for the sensory loss, potentially leading to cognitive decline and psychotic symptoms [5,19]. Social isolation, often experienced by individuals with hearing impairments, exacerbates this risk by amplifying feelings of exclusion and vulnerability [5]. Furthermore, auditory deafferentiation, or the loss of auditory input, alters neural processing and may result in auditory hallucinations [5]. Shared biological factors, such as genetic predispositions or immunological abnormalities, may also underlie the link between hearing loss and psychosis [1,20].

Clinical observations indicate that untreated severe hearing loss can increase the likelihood of psychotic symptoms, including auditory hallucinations and delusions [1,20]. These hallucinations, often described as indistinct or unclear noises, are particularly distressing and may further contribute to delusional thinking and paranoia [1,20]. The progression of psychotic symptoms over time may be compounded by the cognitive and social deterioration associated with hearing impairment [20]. Historical accounts also suggest that auditory hallucinations are commonly reported among individuals with significant hearing loss, underscoring the distressing nature of these experiences [1].

Despite this evidence, some recent studies suggest that the link between hearing loss and psychosis, particularly schizophrenia, may not be as robust as previously thought [1]. While auditory hallucinations remain prevalent in individuals with hearing impairments, the underlying mechanisms remain poorly understood and warrant further research [1,2]. The complexity of this relationship highlights the need for additional investigations to determine causative factors and the interplay between sensory deficits and mental health conditions [5].

Early intervention for hearing loss has the potential to mitigate the severity of associated psychological symptoms [1]. Devices such as hearing aids and cochlear implants not only improve auditory input but also enhance social interaction, which may reduce hallucinations and paranoia [21]. These findings emphasize the importance of prompt treatment to address hearing impairment, thereby alleviating its impact on mental health and improving the overall quality of life [20].

Intervention Implications

Psychotherapy remains a valuable intervention for managing psychiatric comorbidities in individuals with hearing impairment, despite inherent challenges such as communication barriers and difficulty in expressing emotional distress [2]. These challenges often stem from the dual burden of emotional pain and the inability to perceive or articulate therapeutic dialogue effectively [2]. Therapists may experience frustration when communication difficulties arise, underscoring the importance of tailored strategies to overcome these barriers [2]. Certain modalities, such as cognitive behavioral therapy (CBT), have shown particular effectiveness, especially when adapted with visual aids or delivered through sign language [2]. Additionally, group therapy tailored to individuals with hearing loss can foster shared understanding and reduce isolation, further enhancing therapeutic outcomes [2].

Pharmacological interventions, particularly antidepressants, provide another critical avenue for managing psychiatric symptoms in this population [2]. Notably, most psychiatric medications exhibit no significant ototoxicity risk, with exceptions such as certain drugs used for erectile dysfunction, including sildenafil and tadalafil [2]. Emerging evidence suggests a potential role for antidepressants in mitigating auditory damage [2]. For example, doxepin has demonstrated efficacy in reducing neuronal damage in the auditory cortex in animal studies, while findings from human studies remain mixed and require further investigation [2]. Selective serotonin reuptake inhibitors (SSRIs), such as sertraline and escitalopram, and serotonin-norepinephrine reuptake inhibitors (SNRIs), like venlafaxine, have been associated with improvements in mood and anxiety symptoms in individuals with hearing impairment, without evidence of ototoxicity [2]. While more focused research is needed, these classes of antidepressants appear to be safe and beneficial in this population [2].

Technological interventions, including hearing aids and cochlear implants, represent the cornerstone of auditory rehabilitation [4,5]. Cochlear implants, particularly for severe-to-profound hearing loss, have proven effective in enhancing auditory function and quality of life [4,5]. These devices not only improve auditory performance but also decrease the stress and cognitive burden that come with hearing loss [5].

According to studies, those who wear hearing aids report better general mental health, fewer neuropsychiatric difficulties, including agitation, and lower levels of depressive symptoms [10]. In a review of geriatric patients with hearing loss, individuals using hearing aids were found to have a 32% lower prevalence of depressive symptoms and a 28% reduction in neuropsychiatric disturbances such as agitation and irritability compared to those without hearing aids [10]. Another research investigating the effects of cochlear implantation on cognitive decline and quality of life in older adults demonstrated that, after implantation, patients experienced improvements in cognition, speech perception, and overall quality of life [7]. In addition, their depression scores decreased [7]. However, their use is nevertheless uncommon, especially among the elderly and in care facilities [11]. Lack of access to cochlear implants and hearing aids in low- and middle-income nations is one of the main problems [19]. The psychological burden of hearing loss is highest in these areas; therefore, treating hearing impairment might have a major positive impact on the mental health of those who experience it [19].

Emerging regenerative therapies offer hope for reversing hearing loss [2]. Techniques under investigation include gene therapy targeting cochlear hair cell regeneration, stem cell approaches to enhance neural recovery, and pharmacological modulation of key signaling pathways [2]. These innovative strategies hold transformative potential. However, they are still in the early stages of development [2].

Clinicians managing patients with hearing impairment must adopt a multidisciplinary approach, integrating medical, technological, and psychosocial interventions [2]. This includes addressing emergent conditions such as cerumen impaction or sudden sensorineural hearing loss, which require prompt referral to otolaryngologists [2]. Comprehensive care, encompassing auditory rehabilitation programs and tailored psychosocial support, is essential for optimizing both mental health and auditory outcomes in this vulnerable population [2].

Gaps in the Literature

Several critical gaps emerged from this review that warrant future investigation. Although the link between hearing loss and psychiatric disorders such as depression, anxiety, cognitive decline, and psychosis is well-supported, the underlying mechanisms-both neurobiological and psychosocial-remain poorly understood. Longitudinal studies exploring the temporal sequence of hearing loss and psychiatric symptom onset are particularly lacking.

In addition, there is limited research on the effectiveness of tailored psychiatric interventions, such as adapted psychotherapeutic modalities and pharmacological treatments, for individuals with hearing impairment. Data on the comparative benefits and risks of specific antidepressant or antipsychotic drug classes in this population are sparse, and further trials are needed to inform clinical guidelines.

Moreover, there is a need for studies that evaluate integrated care models involving audiologists, psychiatrists, and primary care providers, especially in underserved or low-resource settings. Research focusing on younger individuals with hearing impairment, the impact of early interventions (e.g., cochlear implants), and cultural or geographic disparities in access to care also remains insufficiently explored.

Limitations

Despite the comprehensive nature of this review, several limitations must be acknowledged. First, although we adhered to PRISMA guidelines and conducted a systematic search across three major databases, there remains a possibility of publication bias and the omission of relevant studies not indexed in these databases or published in languages other than English. Second, the heterogeneity among the included studies, ranging in design, population demographics, assessment tools, and outcome measures, limited the ability to conduct a formal meta-analysis or direct comparison across studies. While quality assessment tools such as the NOS and AMSTAR-2 were applied, some included studies still had methodological limitations, including small sample sizes or lack of control for confounders, which may affect the reliability of their findings.

Additionally, as most studies were observational, causal relationships between hearing loss and psychiatric disorders cannot be firmly established. Variability in definitions and measurements of both hearing impairment and psychiatric outcomes further complicates synthesis and interpretation. Lastly, while the review focuses primarily on adult and geriatric populations, evidence pertaining to younger populations with hearing loss remains underrepresented.

## Conclusions

Mental health professionals need to develop greater awareness of "silent" disabilities, such as hearing loss, which can complicate the management of psychiatric disorders. The link between hearing loss and psychiatric conditions is well-documented, with growing evidence suggesting that sensory impairments may contribute to or even trigger these disorders. Psychiatrists, psychologists, and other specialists are uniquely positioned to identify these impairments in the context of psychiatric conditions and refer patients to appropriate services. By understanding the range of available resources, including hearing aids, these professionals can help patients access effective interventions. While significant advancements have been made in both psychiatric care and hearing loss treatment, considerable progress remains in integrating and optimizing these services. It is essential to prioritize early detection and comprehensive intervention to reduce the mental health burden of hearing loss. Addressing both the auditory and psychological aspects of hearing impairment requires a collaborative, multidisciplinary approach involving audiologists, psychologists, and other healthcare providers, particularly in supporting the aging population.

To move the field forward, future research should explore the long-term outcomes of integrated auditory and psychiatric care, identify biomarkers linking sensory and psychiatric decline, and evaluate the effectiveness of emerging interventions such as regenerative therapies. Clinically, there is a need for standardized screening protocols in mental health settings to detect hearing loss early, especially among high-risk populations like the elderly or those with treatment-resistant psychiatric symptoms. Improved training for mental health professionals in recognizing sensory deficits could significantly enhance patient outcomes.
